# Long-term clinical effectiveness of a drug-coated balloon for in-stent restenosis in Femoropopliteal lesions

**DOI:** 10.1186/s42155-021-00205-x

**Published:** 2021-01-11

**Authors:** Kazunori Horie, Akiko Tanaka, Kenji Suzuki, Masataka Taguri, Naoto Inoue

**Affiliations:** 1 Department of Cardiovascular Medicine, Sendai Kousei Hospital, 4-15 Hirose-cho, Aoba-ku, Sendai, Miyagi 980-0873 Japan; 2 Department of Cardiology, Tokyo Saiseikai Central Hospital, Tokyo, Japan; 3 School of Data Science, Yokohama City University, Kanagawa, Japan

**Keywords:** Femoropopliteal segment, In-stent restenosis, Drug-coated balloon, Endovascular treatment

## Abstract

**Background:**

The short-term efficacy of paclitaxel-coated balloons (PCBs) has been established in femoropopliteal in-stent restenosis (ISR) lesions. The aim of this study was to compare 5-year clinical outcomes of patients with femoropopliteal ISR lesions undergoing percutaneous transluminal angioplasty (PTA) with and without PCB.

**Methods:**

After 1:1 propensity score matching, we extracted 50 patients with femoropopliteal ISR lesions undergoing PTA with (*n* = 25) and without (*n* = 25) IN.PACT PCB (Medtronic, Minneapolis, MN) from 106 consecutive ISR patients treated in our hospital between 2009 and 2015. We compared the 5-year outcomes between PCB and non-PCB groups. The primary endpoint was the cumulative 5-year incidence of recurrent restenosis. All-cause mortality, target lesion revascularization (TLR) and unplanned major amputation were also assessed.

**Results:**

The primary patency after PCB treatment at 5 years was significantly higher than the patency after non-PCB treatment (65.7% vs. 18.7%; hazard ratio [HR]: 6.11; 95% confidence intervals [CI]: 2.57–16.82; *p* < 0.001), as well as freedom from TLR (77.6% vs. 53.8%; HR: 3.55; 95% CI: 1.21–12.83; *p* = 0.020). All-cause mortality and unplanned major amputation rates did not significantly differ between the two groups. The Cox proportional hazard multivariate analysis showed that PCB was independently associated with preventing recurrent restenosis (HR: 0.17; 95% CI: 0.06–0.41; *p* < 0.001).

**Conclusions:**

At 5 years, patients with femoropopliteal ISR lesions undergoing PCB treatment showed significantly lower recurrent restenosis than those that underwent non-PCB treatment.

**Evidence-based medicine:**

Level of Evidence: Level 2b, Non-randomized controlled cohort/follow-up study.

## Introduction

Endovascular therapy (EVT) represents an established practice in the treatment of lower extremity peripheral arterial disease (PAD). The use of bare-nitinol stents (BNS) has led to good acute luminal gains in the past 2 decades; however, its primary patency has remained unsatisfactory (Schillinger et al., 2006; Soga et al., 2010; Iida et al., 2014; Tosaka et al., 2012). Several paclitaxel-based devices have been developed to overcome the shortcomings of BNS (Gray et al., 2018; Laird et al., 2019). The new devices are associated with higher primary patency in de novo femoropopliteal lesions than plain balloon angioplasty and BNS, and the recent guideline recommends the primary EVT strategy to be deployed in complex femoropopliteal lesions (Aboyans et al., 2017).

The one-year efficacy of paclitaxel-coated balloons (PCB) has been proven also in treatment of femoropopliteal in-stent restenosis (ISR) after BNS implantation by randomized control trials (RCT) comparing to plain balloon angioplasty; therefore, PCB is one of the best solutions for ISR lesions (Krankenberg et al., 2015; Kinstner et al., 2016; Ott et al., 2017; Cassese et al., 2018). On the other hand, few studies have investigated the long-term patency after PCB treatment in ISR lesions of femoropopliteal segments, and the longest follow-up outcomes have been reported up to 3 years in the previous study (Grotti et al., 2016). In this study, we retrospectively compared the 5-year clinical outcomes of patients with femoropopliteal ISR undergoing percutaneous transluminal angioplasty (PTA) with and without PCB.

## Methods

### Study population

**Fig. 1:**
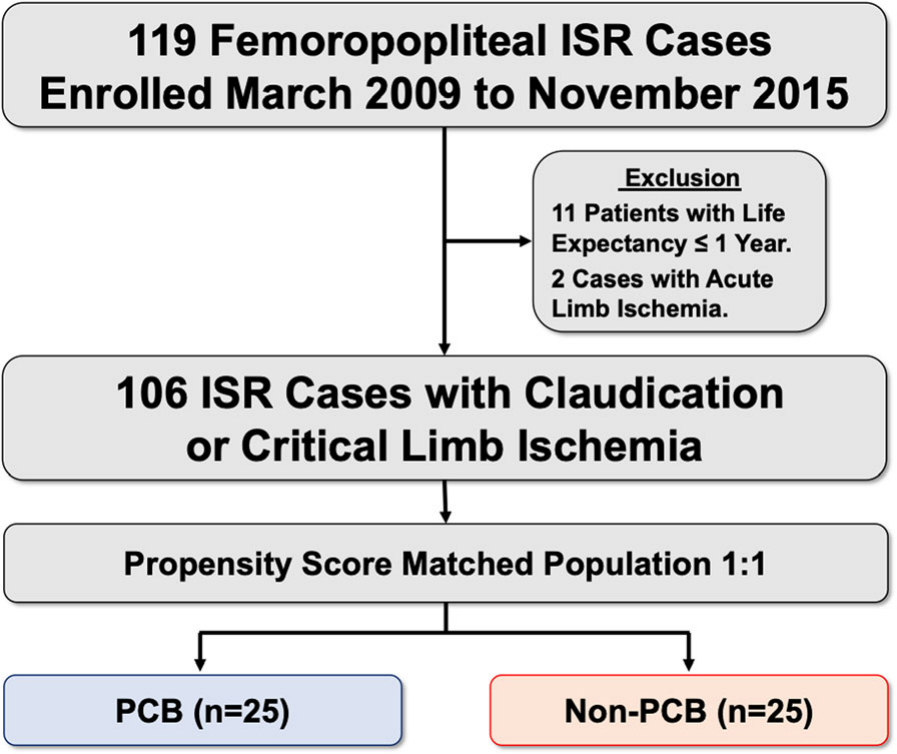
Study Flow chart. ISR indicates in-stent restenosis; PCB, paclitaxel-coated balloon

This retrospective, single-center, non-randomized study was performed to compare the immediate and 5-year outcomes of consecutive patients with femoropopliteal ISR lesions who underwent PTA using PCB (PCB group) and plain balloons (non-PCB group). Given that PCB has been commercially available since December 2018 in our country, we privately imported IN.PACT Pacific PCB (Medtronic, Minneapolis, MN) from 2008 to 2014 after approval by the institutional review board of our hospital. We analyzed 106 consecutive Asian patients (mean age: 72.1 ± 8.7 years; 68 males) with symptomatic PAD who underwent PTA for femoropopliteal ISR lesions at our hospital from 2009 to 2015 (Fig. 1). The key inclusion criteria were age > 50 years, symptomatic PAD (Rutherford category 2 to 5), ISR > 70% at the stented site in femoropopliteal segments (Kinstner et al., 2016). We excluded patients with acute limb ischemia and/or short life expectancy, as in the previous studies (Cassese et al., 2018). The study protocol was developed in accordance with the Declaration of Helsinki and was approved by the institutional review board of our hospital (approval no. 27–33). Informed consent was obtained from all patients.

### Procedures

After local anesthesia with 2.0% xylocaine, a 6.0- or 7.0-French guiding sheath was inserted via the ipsi- or contra-lateral common femoral artery. Unfractionated heparin (5000 IU) was injected initially from the sheath, with an additional 2000 IU given intravenously every hour. In cases of in-stent occlusion, we initially attempted lumen-crossing using 0.018- or 0.014-in. guidewires and microcatheters. If that was unsuccessful, the loop-wire technique was applied, using a 0.035- or 0.018-in. hydrophilic guidewire. If necessary, a retrograde approach was implemented, via either the popliteal or tibial arteries. At first, PTA was performed using plain balloons with nominal diameters same as the reference vessel diameter and with matched length to the lesion’s, evaluated by visual estimation. Balloon dilatation was continued for at least 60 s. IN.PACT PCB was available in nominal diameters of 4 and 6 mm and nominal lengths of 60, 80, and 120 mm in our hospital. When the operators adjudicated that lesions could be covered by one or two PCB with the above sizes after successful balloon dilatation, IN.PACT PCB was dilated for at least 60 s. Inflation time was based on the previous studies conducting PCB treatment (Krankenberg et al., 2015; Fanelli et al., 2014). PCB treatment was used after successful pre-dilatation; therefore, bail-out stenting was not performed in the PCB group.

### Definition and study endpoints

All the lesions were characterized according to the Trans-Atlantic Inter-Society Consensus (TASC) II and Tosaka classification (Tosaka et al., 2012; Eur J Vasc Endovasc Surg, 2007). The immediate success of PTA was defined as achieving any residual stenosis of < 30% of the reference diameter, adjudicated by visual estimation. The Proposed Peripheral Arterial Calcium Scoring System was used to categorize the degree of native femoropopliteal lesion calcification (Rocha-Singh et al., 2014). All angiograms were independently evaluated by two experienced operators for baseline lesion morphology and procedural success. In outpatient follow-up, color Doppler ultrasound assessment was performed routinely every 12 months after EVT to evaluate the patency of the vessel. Restenosis was defined as a peak systolic velocity ratio over 2.4 on duplex ultrasonography, which was considered to indicate > 50% narrowing (Fanelli et al., 2014). The primary study endpoint was the recurrent restenosis within 5 years after PTA for ISR lesions. The secondary endpoints were all-cause mortality, target lesion revascularization (TLR) and unplanned major amputation within 5 years.

### Statistical analysis

In this study, a propensity score (PS) matching analysis was performed to adjust for the differences in baseline clinical characteristics between the two groups. The PS was estimated by a logistic regression model that included patient and lesion characteristics listed in Tables 1 and 2 as exploratory variables. The matching was performed using the nearest-neighbor method, with a caliper of 0.20. Categorical variables were presented as counts (percentages) and compared using the Chi-squared or Fisher’s exact tests. Continuous variables were expressed as mean ± standard deviations and compared using the Student’s t-test or the Mann-Whitney U test based on their distributions. In the matched population, the cumulative incidence of study endpoints was estimated using the Kaplan-Meier method. Hazard ratios (HRs) for recurrent restenosis, all-cause mortality, and TLR were compared between the PCB and non-PCB groups. To identify possible risk factors for recurrent restenosis, HRs for clinically selected patients, lesions, and procedural variables were estimated by univariate and multivariate Cox models. All statistical analyses were performed by two physicians using JMP version 14 (SAS Institute, Cary, NC). Values of *P* < 0.05 were considered statistically significant.

**Table 1:** Baseline Clinical Characteristics

Variables	Overall population			Matched population		
	PCB (***n*** = 32)	Non-PCB (***n*** = 74)	***p***	PCB (***n*** = 25)	Non-PCB (***n*** = 25)	***p***
Age, years*	70.8 ± 7.2	76.7 ± 9.3	0.320	71.9 ± 7.5	71.5 ± 8.9	0.864
Male sex*	20 (62.5)	48 (64.9)	0.816	17 (68.0)	17 (68.0)	1.000
Body mass index, kg/m^2^*	23.8 ± 3.7	23.3 ± 3.5	0.531	23.4 ± 3.7	23.6 ± 2.4	0.770
Hypertension*	31 (96.9)	64 (86.5)	0.167	24 (96.0)	25 (100.0)	1.00
Diabetes mellitus*	26 (81.3)	49 (66.2)	0.163	19 (76.0)	17 (68.0)	0.754
Dyslipidemia*	15 (46.9)	37 (50.0)	0.768	11 (44.0)	13 (52.0)	0.571
Current smoker*	8 (25.0)	15 (20.3)	0.614	5 (20.0)	5 (20.0)	1.000
Chronic kidney disease*	11 (34.4)	32 (43.2)	0.391	9 (36.0)	7 (28.0)	0.762
Hemodialysis*	2 (6.3)	11 (14.9)	0.336	2 (8.0)	2 (8.0)	1.000
Rutherford class*			0.067			1.000
2/3	31 (96.9)	62 (83.8)		24 (96.0)	25 (100.0)	
4	1 (3.1)	7 (9.5)		1 (4.0)	0 (0.0)	
5	0 (0.0)	5 (6.8)		0 (0.0)	0 (0.0)	
Ankle brachial index	0.63 ± 0.12	0.60 ± 0.15	0.375	0.62 ± 0.13	0.61 ± 0.11	0.792
Medication at treatment of ISR						
Aspirin	29 (90.6)	67 (90.5)	1.000	22 (88.0)	24 (96.0)	0.609
P2Y12 antagonist	21 (65.6)	52 (70.3)	0.637	16 (64.0)	13 (52.0)	0.567
Cilostazol	15 (46.9)	30 (40.5)	0.546	13 (52.0)	13 (52.0)	1.000
Oral anticoagulant	2 (6.3)	14 (18.9)	0.140	2 (8.0)	5 (20.0)	0.417

Categorical variables are expressed as number and percentage. Continuous variables are indicated as mean ± SD

*ISR* indicates in-stent restenosis, *PCB* paclitaxel-coated balloon

^*^Variables included in the multivariable analysis to estimate propensity score

**Table 2:** Baseline Lesion Characteristics

Variables	Overall population			Matched population		
	PCB (***n*** = 32)	Non-PCB (***n*** = 74)	***p***	PCB (***n*** = 25)	Non-PCB (***n*** = 25)	***p***
Target lesion						
Superficial femoral artery	32 (100.0)	74 (100.0)	1.000	25 (100.0)	25 (100.0)	1.000
Involving popliteal artery	4 (12.5)	19 (25.7)	0.199	4 (16.0)	7 (28.0)	0.496
Reference vessel diameter, %	5.65 ± 0.40	5.54 ± 0.47	0.282	5.61 ± 0.37	5.50 ± 0.49	0.367
Lesion length of native disease, %	223.4 ± 68.4	200.9 ± 81.4	0.173	222.0 ± 73.5	209.6 ± 78.4	0.567
Total occlusion of native disease, %	27 (84.4)	55 (74.3)	0.318	20 (80.0)	21 (84.0)	1.000
TASC II classification of native disease			0.177			0.217
A/B	4 (12.5)	21 (18.4)		3 (12.0)	6 (24.0)	
C	6 (18.8)	15 (20.3)		5 (20.0)	5 (20.0)	
D	22 (68.7)	38 (51.3)		17 (68.0)	14 (56.0)	
PACSS classification of native disease			0.937			0.702
0–3	27 (84.4)	64 (86.5)		20 (80.0)	22 (88.0)	
4	5 (15.6)	10 (13.5)		5 (20.0)	3 (12.0)	
Types of ISR-related stents			1.000			1.000
Bare-nitinol stent	30 (93.7)	69 (93.2)		24 (96.0)	25 (100.0)	
SMART™	10 (31.3)	21 (28.4)		7 (28.0)	9 (36.0)	
Luminexx™	15 (46.9)	33 (44.6)		14 (56.0)	13 (52.0)	
Zilver 518™	1 (3.1)	3 (4.1)		1 (4.0)	0 (0.0)	
Misago™	4 (12.5)	11 (14.9)		2 (8.0)	2 (8.0)	
LIFE STENT™	0 (0.0)	1 (1.4)		0 (0.0)	1 (4.0)	
Drug-coated stent (Zilver PTX™)	2 (6.3)	5 (6.8)		1 (4.0)	0 (0.0)	
ISR pattern*			< 0.001			0.581
Focal (≤50 mm)	2 (6.3)	25 (33.8)		2 (8.0)	2 (8.0)	
Diffuse (> 50 mm)	24 (75.0)	22 (29.7)		19 (76.0)	16 (64.0)	
Occlusion	6 (18.7)	27 (36.5)		4 (16.0)	7 (28.0)	
ISR length*	120.3 ± 59.3	131.1 ± 95.6	0.555	126.0 ± 57.4	134.4 ± 71.4	0.649
Distal run-off vessel			0.378			1.000
2/3	23 (71.9)	45 (60.8)		17 (68.0)	18 (72.0)	
0/1	9 (28.1)	29 (39.2)		8 (32.0)	7 (28.0)	
Stent fracture classification			0.582			1.000
0/1/2	30 (93.7)	72 (97.3)		22 (92.0)	24 (96.0)	
≥ 3	2 (6.3)	2 (2.7)		2 (8.0)	1 (4.0)	

Categorical variables are expressed as number and percentage. Continuous variables are indicated as mean ± SD

*ISR* indicates in-stent restenosis, *PACSS* Proposed Peripheral Arterial Calcium Scoring System, *PCB* paclitaxel-coated balloon, *TASC* Trans-Atlantic Inter-Society Consensus

^*^Variables included in the multivariable analysis to estimate propensity score

## Results

### Study population

After PS matching, the final study population consisted of 25 matched patients in each group (Fig. 1).

### Baseline clinical characteristics

Baseline patient and lesion characteristics before and after PS matching are displayed in Tables 1 and 2. Before PS matching, no significant differences in baseline clinical characteristics were observed between PCB and non-PCB groups, except for ISR patterns. After PS matching, baseline patient and lesion characteristics were well balanced between the two groups. Table 3 shows the comparison of procedural characteristics and results. Before and after PS matching, the balloon sizes and pressure before PCB dilatation were similar between the two groups. Since PCB was used after successful balloon pre-dilatation, the patients in the PCB group did not undergo bail-out stenting. The bail-out stenting was performed in 20 patients of the original population in the non-PCB group and the 12 patients had Tosaka III lesions (60.0%). The PCB dilatation was successful in all patients of the PCB group. There was no significant difference in the rates of complications between the two groups.

**Table 3:** Procedural Results

Outcomes	Overall population			Matched population		
	PCB (***n*** = 32)	Non-PCB (***n*** = 74)	***p***	PCB (***n*** = 25)	Non-PCB (***n*** = 25)	***p***
Pre-dilatation	32 (100.0)	74 (100.0)	1.000	25 (100.0)	25 (100.0)	1.000
Balloon diameter of pre-dilatation, (mm)	5.27 ± 0.54	5.31 ± 0.64	0.729	5.22 ± 0.46	5.44 ± 0.56	0.137
Balloon/artery ratio of pre-dilatation	0.94 ± 0.11	0.96 ± 0.12	0.307	0.93 ± 0.10	0.99 ± 0.11	0.052
Balloon pressure of pre-dilatation, (atm)	11.5 ± 3.3	11.1 ± 3.0	0.526	11.5 ± 3.5	10.8 ± 2.7	0.441
The detail of PCB						
Diameter, (mm)	5.63 ± 0.79	–	–	5.60 ± 0.82	–	–
Total length, (mm)	133.8 ± 52.7	–	–	137.6 ± 52.1	–	–
Inflation time, (sec)	72.2 ± 32.2	–	–	74.4 ± 35.8	–	–
Dilatation pressure, (atm)	9.1 ± 3.1	–	–	9.5 ± 3.4	–	–
Bail-out stenting	0 (0.0)	20 (27.0)	< 0.001	0 (0.0)	7 (28.0)	0.001
Complications						
Slow flow	3 (9.4)	5 (6.8)	0.695	2 (4.0)	1 (3.0)	1.000
Distal embolization	2 (6.3)	3 (4.1)	0.637	2 (4.0)	0 (0.0)	0.490
Vessel perforation	0 (0.0)	0 (0.0)	1.000	0 (0.0)	0 (0.0)	1.000

Categorical variables are expressed as number and percentage. Continuous variables are indicated as mean ± SD

*PCB* indicates paclitaxel-coated balloon

### Clinical outcomes

**Fig. 2:**
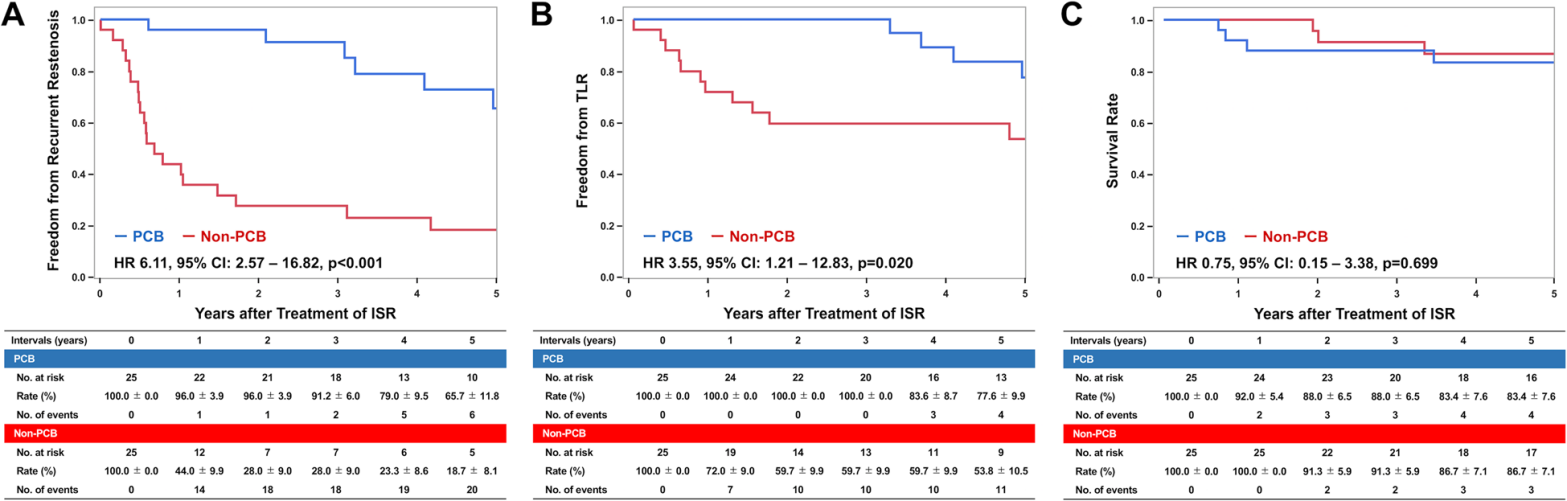
Clinical Events After EVT within 5 Years. a freedom from recurrent restenosis, **b** freedom from TLR, and **c** survival rate. CI indicates confidence intervals; HR, hazard ratio; ISR, in-stent restenosis; PCB, paclitaxel-coated balloon; TLR, target lesion revascularization

**Fig. 3:**
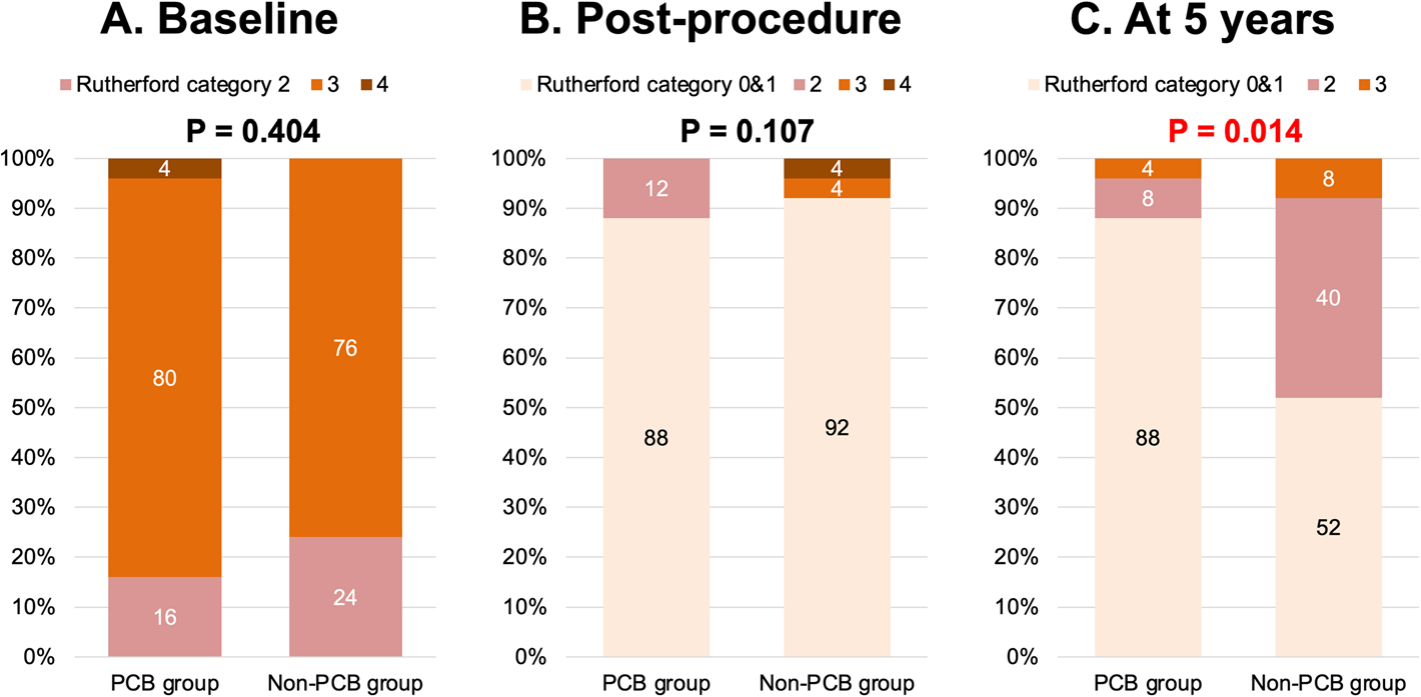
Change in Rutherford Category before and after Procedure, and at 5-year Follow-up in the Matched Population. **a** The Rutherford categories before and **b** after the percutaneous transluminal angioplasty were not difficult between the PCB and non-PCB groups. **c** At 5 years, the rate of patients with Rutherford category 0 & 1 was significantly higher in the PCB group (*p* = 0.014). PCB indicates paclitaxel-coated balloon

Five-year follow-up information was obtained for 19 (76.0%) PCB patients and in 20 (80.0%) non-PCB patients (*p* = 1.000) after PS matching. At 5 years, the rate of freedom from recurrent restenosis was significantly higher in the PCB group (65.7%) than that in the non-PCB group (18.7%) with a HR of 6.11 and 95% confidence interval (CI) of 2.57–16.82 (*p* < 0.001; Table 4 and Fig. 2), as well as the rate of freedom from TLR (77.6% vs. 53.8%; HR: 3.55; 95% CI: 1.21–12.83; *p* = 0.020). The Rutherford category was improved in both groups similarly after the procedure; whereas the rate of patients with Rutherford category 0 and 1 was significantly higher in the PCB group at 5 years (*p* = 0.014; Fig. 3). There was no significant difference between the two groups in terms of all-cause mortality (16.0% vs. 12.0%; HR: 1.34; 95% CI: 0.30–6.81; *p* = 0.699) (Table 4). No unplanned major amputation was observed in any patient of the two groups. The Cox proportional hazard multivariate analysis revealed that the use of PCB was independently associated with a lower incidence of recurrent restenosis (HR 0.17, 95% CI: 0.06–0.41; *p* < 0.001) (Table 5).

**Table 4:** Clinical Events after Treatment for In-stent Restenosis at 5 years

Variables	Overall population			Matched population		
	PCB (***n*** = 32)	Non-PCB (***n*** = 74)	***p***	PCB (***n*** = 25)	Non-PCB (***n*** = 25)	***p***
Recurrent ISR at 5 years	8 (25.0)	53 (71.6)	< 0.001	6 (24.0)	20 (80.0)	< 0.001
All-cause death at 5 years	4 (12.5)	9 (12.2)	0.963	4 (16.0)	3 (12.0)	0.699
Target lesion revascularization at 5 years	5 (15.6)	30 (40.5)	0.006	4 (16.0)	11 (44.0)	0.020
Unplanned major amputation at 5 years	0 (0.0)	1 (1.4)	0.491	0 (0.0)	0 (0.0)	1.000

Categorical variables are expressed as number and percentage, and are calculated based on Kaplan-Meier estimate

*ISR* indicates in-stent restenosis, *PCB* paclitaxel-coated balloon

**Table 5:** Predictors of Recurrent Restenosis within 5 Years after Treatment of In-stent Restenosis in the Matched Population

Variables	Univariable			Multivariable		
	HR	95% CI	*p*	HR	95% CI	*p*
Age, (per 1 year)	1.03	0.92–1.03	0.343			
Male sex	1.40	0.60–3.55	0.460			
Body mass index (per 1.0)	0.99	0.89–1.10	0.835			
Dyslipidemia	0.83	0.37–1.80	0.640			
Diabetes mellitus	1.24	0.51–2.77	0.618			
Current smoker	1.58	0.61–3.64	0.324			
Chronic kidney disease	1.52	0.65–4.16	0.351			
Hemodialysis	1.12	0.18–3.78	0.881			
Use of cilostazol	1.30	0.60–2.91	0.505			
Involvement of popliteal lesion	1.51	0.59–3.44	0.370			
Reference vessel diameter (per 1.0 mm)	0.96	0.40–2.43	0.925			
ISR length (per 10.0 mm)	1.04	0.98–1.10	0.172	1.04	0.97–1.10	0.251
Tosaka type III (occlusion)	2.10	0.85–4.73	0.102	1.39	0.55–3.23	0.463
PACSS grade 4 of native lesions	1.05	0.40–3.58	0.933			
Tibial runoff ≤1	1.65	0.72–3.61	0.224			
Use of PCB at ISR treatment	0.16	0.06–0.39	< 0.001	0.17	0.06–0.41	< 0.001

*CI* indicates confidence interval, *HR* hazard ratio, *ISR* in-stent restenosis, *PACSS* Proposed Peripheral Arterial Calcium Scoring System, *PCB* paclitaxel-coated balloon

## Discussion

The main findings of the present study are as follows: (1) the recurrent restenosis rate at 5 years after PCB treatment was significantly lower than that after non-PCB treatment; (2) the Cox multivariate analysis revealed that the use of PCB significantly reduced the incidence of recurrent restenosis; and (3) the cumulative rates of procedural complications, all-cause mortality, and major amputation were not different between the two groups.

Balloon angioplasty can potentially injure the vessel due to overstretching of the wall, denudation of endothelium, rupture of internal elastic lamina, and medial tear leading to the stimulation of smooth muscle cells; therefore, paclitaxel plays an important role in suppressing the stenotic processes (Yahagi et al., 2014). Restenosis and de novo femoropopliteal plaques are different in cellular composition and cell proliferation, the former being highly cellular and comprised primarily of smooth muscle cells (Johnson et al., 1990; Edlin et al., 2009). The suppression of neointimal growth through the antiproliferative effect of paclitaxel led to a consistent lower risk for repeat revascularization and recurrent restenosis (Krankenberg et al., 2015; Kinstner et al., 2016; Ott et al., 2017; Cassese et al., 2018). Thus, the recent guidelines recommend PCB treatment rather than plain balloon angioplasty in ISR lesions, as class IIb (Aboyans et al., 2017).

RCTs reported that the primary patency rates at 6 months after PCB treatment were significantly higher than those after plain balloon angioplasty. However, the 6-month patency after PCB treatment ranged from 58.8 to 84.6%, according to ISR complexity such as lesion length and total occlusion; whereas the 6-month patency in the control group treated by plain balloon angioplasty was reported to range from 41.3% to 55.3% and it could not differ with the lesion complexity (Krankenberg et al., 2015; Kinstner et al., 2016; Ott et al., 2017; Cassese et al., 2018). The difference between the patency rates might be due to the difficulty associated with vessel preparation before PCB treatment. This study included patients with mainly Tosaka I and II lesions (84.0%) of relatively short length (126 ± 57.4 mm); therefore, the patency rate after PCB treatment was high at 96.0% within one year, comparing to that in the previous reports (Krankenberg et al., 2015; Kinstner et al., 2016; Ott et al., 2017; Cassese et al., 2018).

In the present study, the rate of Tosaka I and III lesions was significantly higher in the original non-PCB group before the matching. The possible reason of the discrepancy was the difference of the procedural success and the patency rate according to the severity of ISR patterns. The freedom from recurrent occlusion after plain PTA in Tosaka I ISR was reported to be 84.1% at 3 years; therefore, we might not tend to use PCB for focal ISR (Tosaka et al., 2012). On the other hand, in-stent occlusion was generally more difficult to achieve successful pre-dilatation (Grotti et al., 2016) and the bail-out stenting was performed in mainly Tosaka III in-stent occlusion in the present study. Consequently, the rate of patients with Tosaka III was significantly lower in both the original and matched PCB group. Moreover, the high rate of patients with stent-in-stent treatment might influence of the poor patency rate in the non-PCB group.

As a previous report demonstrated that the patency after PCB was lower in Tosaka III lesions than in the others (Grotti et al., 2016), it might be better to observe patients carefully after stenting in femoropopliteal lesions and to treat early-staged ISR lesions using PCB. On the other hand, the current meta-analysis demonstrated that debulking devices improved the patency after PTA in patients with complex ISR lesions (Li et al., 2020). Particularly, a combination of laser atherectomy (LA) and PCB was reported to be more effective in reducing the TLR rate within 2 years in Tosaka II and III ISR lesions than LA and plain balloon angioplasty (Kokkinidis et al., 2020; van den Berg et al., 2014). In PCB treatment, lesion modification might be effective in overcoming the complex femoropopliteal ISR lesions.

Liistro et al. demonstrated that PCB reduced the rates of recurrent restenosis and TLR within one year significantly more than plain balloon angioplasty in ISR patients with diabetes and a high prevalence of critical limb ischemia (Liistro et al., 2014); however, they showed that the benefit provided by PCB was not evident at 3 years (Grotti et al., 2016). On the other hand, although the patency after PCB treatment decreased gradually from 96.0% at 2 years to 65.7% at 5 years in this study, the patency rate was superior at 5 years to that after plain PTA treatment significantly. The possible reasons of the low rate of this phenomenon, known as “late catch-up,” might be due to the difference of patient characteristics. As mentioned above, the PCB group of this study included mainly claudicants and Tosaka II ISR lesions rather than the previous study which had patients with complex characteristics such as diabetes (100.0%), critical limb ischemia (75.0%) and Tosaka III lesions (51.0%) (Grotti et al., 2016; Liistro et al., 2014). These characteristics were associated with poor long-term patency after PCB treatment in patients with de novo femoropopliteal lesions (Laird et al., 2019); therefore, the adverse conditions of PAD patients might influence the patency also in ISR lesions.

The results of a recent meta-analysis aroused concern about an increased risk of death associated with the use of paclitaxel-based devices in lower-limb EVT for PAD (Katsanos et al., 2018). Whereas the latest large-scale RCT comparing paclitaxel-coated and uncoated devices demonstrated that use of coated devices did not increase the mortality of PAD patients within 4 years (Nordanstig et al., 2020). The concern about safety of paclitaxel-coated devices is still inconclusive. Although this study was retrospective and included quite small number of PAD patients, there was not difference in mortality within 5 years between patients treated with and without PCB.

### Study limitations

This study has several limitations. First, it was a single-center trial with a small sample size. Therefore, the shortcoming of this study was small number of patients to support meaningful statistics, and especially a valid multivariable analysis. Moreover, this was retrospective; therefore, the follow-up rate was not high. Accordingly, outcomes should be read with extreme cautiousness. Second, because the study duration was 6 years, there was a possibility of bias over time in deciding the EVT procedure. Third, the endpoints were adjudicated by independent observers but not by an external core laboratory. Finally, although we performed PS matching to adjust for the differences in baseline clinical and procedural characteristics between the two groups, potential bias could not be excluded in this study, and might have affected the conclusions.

## Conclusions

At 5 years, patients with femoropopliteal ISR lesions treated through PCB showed significantly lower recurrent restenosis and TLR rates than those who underwent non-PCB treatment.

## Data Availability

Please contact author for data requests.

## Abbreviations

BNS: Bare-nitinol stent
CI: Confidence interval
EVT: Endovascular therapy
HR: Hazard ratio
ISR: In-stent restenosis
LA: Laser atherectomy
PACSS: Proposed Peripheral Arterial Calcium Scoring System
PAD: Peripheral arterial disease
PCB: Paclitaxel-coated balloon
PS: Propensity score
PTA: Percutaneous transluminal angioplasty
TASC: Trans-Atlantic Inter-Society Consensus
TLR: Target lesion revascularization
